# Scraps

**Published:** 1889-03

**Authors:** 


### SCRAPS

PICKED UP BY THE ASSISTANT-EDITOR,

**Too Knowing.**—Pompous physician (to patient's wife): "Why did you delay sending for me until he was out of his mind?" Wife: "Oh, doctor, while he was in his right mind he wouldn't let me send for you."—*The Medical and Surgical Reporter.*

**Un dentiste qui a dû être vexé.**—Une jeune dame souffrait affreusement des dents. Elle va chez un dentiste qu'elle ne connaissait pas et le prie de lui arracher la canine malade. L'affaire faite, dit le *Figaro*, elle place une pièce de 10 francs dans la main de l'opérateur. Celui-ci, trouvant ses honoraires un peu minces et regardant la pièce dédaigneusement: "Ceci est-il pour mon domestique?" dit-il. "Non, Monsieur," reprend la dame avec son plus gracieux sourire, "c'est pour vous deux!"—*L'Union médicale.*

"**An Imperfect (?) Form of Massage.**"—Referring to "A Full Account of the System of Friction as adopted and pursued with the greatest success in cases of Contracted Joints and Lameness from various causes, by the late eminent surgeon, John Grosvenor, Esq., of Oxford, with observations on those cases to which it is most applicable by William Cloebury, Member of the Royal College of Surgeons of London, and one of the surgeons to the Radcliffe Infirmary, Oxford," Dr. William Murrell (*Masso-Therapeutics*, Fourth Edition, 1889, pp. 199—201) says: "Although published as recently as 1825, it is a comparatively rare work, there not being a copy in any of the medical libraries in London. . . . I cannot gather from the work itself whom he employed to carry out the requisite manipulations, but the following extract from 'The Black Mousquetaire' throws some light on the subject:—

'Oh, woman!' Sir Walter observes, 'when the brow
's wrung with pain, what a minist'ring Angel art thou!'
Thou'rt a 'minist'ring Angel' in no less degree,
I can boldly assert, when the pain's in the knee:
And medical friction Is, past contradiction,
Much better perform'd by a She than a He.
A fact which, indeed, comes within my own knowledge,
For I well recollect, when a youngster at College,
And therefore can quote A surgeon of note,
Mr. Grosvenor of Oxford, who not only wrote
On the subject a very fine treatise, but, still as his
Patients came in, certain soft-handed Phyllises
Were at once set to work on their legs, arms and backs,
And rubb'd out their complaints in a couple of cracks."

**Puerperal Self-possession.**—When the Duchesse de Berri was delivered of the child which she added to the house of her murdered husband and to the race of the Bourbons, she exhibited in a remarkable manner the determination that afterwards characterised her in more public incidents. Her labour was unusually easy and quick, and consequently the child was born before the arrival of the dignitaries of the Court, whose presence was, by Royal custom, required for authentication of the parentage and sex of the infant. This custom was more than a mere formality in the Duchess's case; for Napoleonists and Republicans had circulated a scandalous tale that her pregnancy was a got-up affair for the purpose of imposing a foundling upon France. The Duchess, therefore, would not allow the umbilical cord to be cut till one of the Court officials should appear; and on the arriva of the marshal-in-waiting, she said to him, "Approchez, M. le Maréchal! Vous voyez l'enfant et moi, que nous ne sommes qu'un!"—*Life of Sir Robert Christison.*

**Clifton Described by an Outsider.**—Nobody drinks the Hotwells water now. The very pump-room, which overlooked the dreary oasis of mud spanned by the beautiful Suspension Bridge, has long been removed for the very sufficient reason that the money taken for the use of the excellent swimming bath and (the once famed) mineral water, did not pay even the outgoing expenses. For this state of things Clifton has itself to thank. Local jealousies, an utter absence of the spirit of co-operation and enterprise among its inhabitants repel and drive away strangers. Unlike Bath, even in the days of its prosperity Clifton was as dull as ditchwater, very nearly as dull as its modern representative, if dulness of so impenetrable a character can ever hope to be equalled. With beauties of situation and scenery which render it naturally attractive, Clifton, owing to the exclusiveness of its *parvenu* aristocracy, ridiculed by Sheridan in *The School for Scandal*, combined with the absence of any sort of amusement, is the last place to which a fashionable doctor would be mad enough to consign a consumptive patient. For the past thirty years the place has been the paradise of a class of German street musicians, who would be driven headlong out of London—of screaming "hooters"—of nursery-maids afflicted with incurable melancholy; but, with the exception of those, whose suicidal tendencies draw them in the direction of the Suspension Bridge, which the trustees wisely keep unprotected for their accommodation, no strangers visit Clifton except for the purpose of leaving it with as little delay as possible.—*Doctors* AND *Doctors*, 1888.

---

**Important to Subscribers to this Journal.**—A "Reader" who, except a disregard of spelling, gives me nothing beyond the Bath postmark by which to identify him, writes to me in reference to the printed letter and form enclosed with the number for last December. The part about which a "Reader" writes was worded thus: "As the Society under whose auspices the *Journal* is published is not running it to make money, will you do me the kindness to get the accompanying form filled up by one who is not yet a Subscriber?" The "Reader" says this is "a very good reason why you should supply the Journal gratis to me, but a very poor one why I should get another suscriber" *(sic).*

I feel sure that the intelligence of Bath will not be willing to accept a "Reader" as its representative. But in case there should, there or elsewhere, be any dull person who stands in need of enlightenment, I will ask him to be so good as to insert in the letter, between "make" and "money," the words "or lose." Then I trust that my anonymous correspondent and others will see that it is their duty, by extending the circulation of the *Journal*, to secure from pecuniary loss those who, in the interests of science, took upon themselves its responsibility.

I felt no hesitation in making my appeal, because I was not asking others to help forward a commercial venture out of which the Society wanted "to make money," but only seeking their co-operation in this effort to supply the profession of the West of England and South Wales with a periodical of its own—an effort which has been enthusiastically and successfully supported, and which certainly deserves to meet with all the encouragement the doctors of the district can give it.

I regret that I have no power to supply a "Reader" with "the Journal gratis;" but if he will give me his name and address, I will ask the Managing Committee to do so. Such unexampled dulness deserves a prize.

